# Exploring cerebellar transcranial magnetic stimulation in post-stroke limb dysfunction rehabilitation: a narrative review

**DOI:** 10.3389/fnins.2025.1405637

**Published:** 2025-02-03

**Authors:** Zhan Wang, Likai Wang, Fei Gao, Yongli Dai, Chunqiao Liu, Jingyi Wu, Mengchun Wang, Qinjie Yan, Yaning Chen, Chengbin Wang, Litong Wang

**Affiliations:** ^1^Rehabilitation Medicine Department, The Second Hospital of Dalian Medical University, Dalian, China; ^2^Rehabilitation Center, Qilu Hospital of Shandong University, Jinan, China; ^3^University of Health and Rehabilitation Sciences, Qingdao, China; ^4^Department of Neurology, Dalian Municipal Central Hospital, Dalian, China

**Keywords:** cerebellar transcranial magnetic stimulation, stroke rehabilitation, limb dysfunction, neuroplasticity, motor recovery

## Abstract

This review delves into the emerging field of cerebellar Transcranial Magnetic Stimulation (TMS) in the rehabilitation of limb dysfunction following a stroke. It synthesizes findings from randomized controlled trials and case studies, examining the efficacy, safety, and underlying mechanisms of cerebellar TMS. The review outlines advancements in TMS technologies, such as low-frequency repetitive TMS, intermittent Theta Burst Stimulation, and Cerebello-Motor Paired Associative Stimulation, and their integration with physiotherapy. The role of the cerebellum in motor control, the theoretical underpinnings of cerebellar stimulation on motor cortex excitability, and the indirect effects on cognition and motor learning are explored. Additionally, the review discusses current challenges, including coil types, safety, and optimal timing and modes of stimulation, and suggests future research directions. This comprehensive analysis highlights cerebellar TMS as a promising, though complex, approach in stroke rehabilitation, offering insights for its clinical optimization.

## 1 Introduction

Stroke remains a preeminent cause of chronic disability on a global scale, severely affecting countless individuals annually (Dhuri et al., 2021). It is primarily characterized by an abrupt interruption of blood flow to a specific brain region, resulting in neuronal damage (Premilovac et al., 2020). Such cerebral ischemic events frequently lead to extensive physical disabilities, with limb dysfunction being notably incapacitating (McHutchison et al., 2019). This diminution in limb capability not only markedly deteriorates a patient’s life quality but also imposes substantial dependence on caregivers and a consequent societal burden.

Traditional Transcranial Magnetic Stimulation (TMS), focusing primarily on the primary motor cortex (M1) for upper limb rehabilitation, has demonstrated potential in stroke recovery. However, its effectiveness in ameliorating lower limb dysfunction and balance issues is somewhat constrained. This limitation largely stems from the inadequate penetration depth of the standard figure-8 coil, which often fails to sufficiently stimulate lower limb regions of the M1 (Zhao et al., 2019). Moreover, patient-specific response variability and the extent of neural damage further limit the efficacy of conventional TMS (Pasley et al., 2009). As a result, recent researches have begun exploring stimulation of non-motor cortical areas, such as the prefrontal cortex, and non-cortical areas like the cerebellum, in search of more effective rehabilitation strategies. A distinctive feature of cerebellar TMS is its capacity to activate motor-related cortical and subcortical regions, including the thalamus, M1, posterior parietal cortex (PPC), and premotor cortices (PMC) (Casula et al., 2016). Such unique activation leverages the cerebellum’s intrinsic capability for motor adaptation and integration, thus offering an innovative approach in addressing post-stroke limb dysfunction. Additionally, cerebellar TMS has been linked to changes in resting-state functional connectivity of brain networks, showing promise in rehabilitation and motor learning processes (Dum and Strick, 2003; Spampinato D. A. et al., 2020; Xia et al., 2022). Emerging research on cerebellar TMS distinguishes it as a forward-looking and promising therapeutic avenue, potentially exceeding traditional methods in targeting deeper motor areas and efficaciously managing lower limb and balance dysfunctions.

The purpose of this review is to meticulously explore and synthesize the current research on cerebellar TMS as it pertains to limb dysfunction post-stroke. By examining a spectrum of studies, ranging from randomized clinical trials to case reports, this review aims to present a comprehensive overview of the efficacy, safety and mechanisms of action of cerebellar TMS. Furthermore, it seeks to highlight the challenges and limitations encountered in the current research landscape, while also identifying future directions that could contribute to the optimization of this therapeutic intervention in clinical practice.

## 2 Background

### 2.1 The evolution and theoretical foundations of TMS in stroke rehabilitation

TMS, a non-invasive cerebral stimulation modality, has undergone significant advancements since its inception in 1985 (Barker et al., 1985). TMS employs magnetic fields to generate electric currents within specific cerebral regions by positioning a magnetic coil close to the scalp (Gilbert et al., 2019). Activation of this coil induces a magnetic pulse that penetrates the skull, subsequently eliciting a minor electric current in the brain tissue beneath (Schluter et al., 2019). The impact of this current on neuronal functioning varies according to the frequency of stimulation: high-frequency TMS (exceeding 5 Hz) tends to augment cortical excitability, whereas low-frequency TMS (1 Hz or below) is associated with reduced excitability (Zhang et al., 2022).

A critical development in TMS technology is Theta Burst Stimulation (TBS), an time-effective stimulation mode that can accomplish in mere minutes what conventional repetitive TMS (rTMS) achieves in 20–30 min (Corp et al., 2020). TBS is categorized into two forms: continuous TBS (cTBS), which yields prolonged inhibitory effects on the cerebral cortex, and intermittent TBS (iTBS), known for inducing long-term potentiation (LTP) like effects when applied to the cerebral and cerebellar cortex. This form of stimulation bolsters neuroplasticity in the cerebral cortex and has demonstrated efficacy in enhancing motor function recovery in stroke survivors, with benefits persisting for at least 30 min post-stimulation (Pauly et al., 2021).

The integration of TMS into stroke rehabilitation has progressively evolved, initially focusing on the M1 for motor restoration. Subsequent investigations expanded its application to encompass modulation of other cerebral regions, notably the cerebellum. The advent of diverse TMS protocols, including rTMS and iTBS, has refined clinical applications, facilitating more targeted interventions for an array of stroke-related impairments.

The advancement of TMS in stroke rehabilitation is anchored in three principal theoretical frameworks concerning limb recovery. The compensation model posits that recovery involves engaging alternative neural pathways to offset those impaired by stroke (Jaillard et al., 2005). The interhemispheric competition model hypothesizes a post-stroke imbalance in excitatory and inhibitory interactions between the brain’s hemispheres affecting motor functions, which TMS can modulate (Grefkes and Fink, 2014). Finally, the “biphasic balance” recovery model posits a two-phase recovery process, commencing with interhemispheric inhibition and evolving into a balanced bilateral activation (Di Pino et al., 2014). These theoretical frameworks offer valuable insight for the application of TMS in neural rehabilitation, guiding the refinement and optimization of TMS protocols for enhancing functional outcomes post-stroke.

Paired Associative Stimulation (PAS) is a specialized variant of paired-pulse transcranial magnetic stimulation (ppTMS). Unlike traditional TMS, which typically involves direct stimulation of the motor cortex or other brain regions (Derosiere et al., 2022), PAS combines a TMS pulse directed at a cortical area with peripheral nerve electrical stimulation. This approach leverages the temporal relationship between sensory input and cortical excitability (Stampanoni Bassi et al., 2020). This method is grounded in the Hebbian plasticity model (Shikauchi et al., 2023), where the sequence and timing of stimuli can induce LTP or long-term depression (LTD) of synaptic connections. Specifically, when the peripheral stimulus precedes cortical stimulation, LTP is induced, promoting synaptic strengthening, whereas the reverse sequence leads to LTD, which weakens synaptic connections (Chindemi et al., 2022). This dynamic interaction facilitates cortical plasticity, which is essential for motor recovery following neurological injuries. Furthermore, PAS can also involve cortical–cortical stimulation using a double-coil setup to target different brain regions, thereby enhancing the precision and efficacy of neuromodulation. Clinically, PAS has demonstrated promise in treating conditions such as stroke, Parkinson’s disease, and chronic pain, with evidence indicating improvements in motor performance and neuroplasticity (Shikauchi et al., 2023). In the context of stroke rehabilitation, PAS is emerging as a key mechanism for enhancing motor recovery, with the potential to synergize with other neuromodulatory interventions, such as cerebellar TMS, to further promote cortical reorganization and functional recovery.

### 2.2 Role of the cerebellum in motor control

Traditionally, the cerebellum has been acknowledged for its critical role in refining motor actions and maintaining balance and coordination (Mirdamadi and Block, 2021). This perception, while accurate, only partially represents the cerebellum’s complex involvement in motor control. Contemporary neuroscience has substantially expanded our understanding, revealing the cerebellum’s integral role in a myriad of motor and cognitive functions (De Doncker et al., 2021).

Recent advancements in research have elucidated the cerebellum’s expansive role in motor control, highlighting a complex network of connections extending well beyond mere coordination. Cerebro-cerebellar loops, for instance, establish links between the cerebellum and the cerebral cortex (Caligiore et al., 2017; Spampinato D. et al., 2020; Carey, 2024). These connections facilitate intercommunication between the cerebellum and brain regions involved in motor planning, such as the prefrontal cortex, execution (like the M1), and sensory processing (Zhang et al., 2019; Carey, 2024). The cerebellar-thalamic-cortical pathway, pivotal in modulating motor commands, is instrumental for motor learning (Mawase et al., 2017). It enables the cerebellum to fine-tune and adapt motor actions (Hirjak et al., 2020), a capability critical for acquiring new motor skills or reacquiring skills after a stroke. Furthermore, the cerebellum’s integration with the vestibular system through vestibulo-cerebellar connections is essential for balance and spatial orientation (Pushchina et al., 2022). Recent studies (Blatt et al., 2013; Ferrari et al., 2022) also suggest the presence of limbic-cerebellar connections, indicating the cerebellum’s involvement in the emotional dimensions of movement. Additionally, spino-cerebellar tracts relay essential sensory feedback from the spinal cord regarding the state of muscles and joints (Baek et al., 2019), further enhancing the cerebellum’s ability for precise motor control. Collectively, these pathways highlight the cerebellum’s comprehensive role in the adaptive control of movement, a factor of considerable relevance in formulating targeted strategies for stroke rehabilitation.

In the context of stroke recovery, the cerebellum’s role in adaptive motor control assumes significant importance. Moreover, contemporary research is delving into the cerebellum’s involvement in the higher-order processing of motor tasks, suggesting its participation in the cognitive aspects of motor planning and decision-making. This evolving perspective accentuates the cerebellum’s potential as a therapeutic target in stroke rehabilitation, extending beyond the traditional focus on the M1.

### 2.3 Molecular pathways activated by cerebellar TMS in synaptic plasticity

Cerebellar TMS is a powerful neuromodulatory tool that induces plasticity within the cerebellum, a brain region crucial for motor control, cognitive processing, and emotional regulation. Key molecular mechanisms involved in cerebellar plasticity during TMS include glutamatergic signaling, calcium signaling, gamma-aminobutyric acid (GABA) pathways, Brain-Derived Neurotrophic Factor (BDNF) upregulation, and cAMP/PKA pathways.

Purkinje cells in the cerebellum play a crucial role in inhibiting cerebellar neuronal activity through the neurotransmitter GABA. Cerebellar TMS of the cerebellum can modulate the activity of these Purkinje cells, thus influencing cortical cerebellar inhibition (CBI) (Spampinato et al., 2021). Notably, GABA-B receptors, which are the most widely distributed inhibitory receptors in the cerebellum, are involved in mediating both immediate and long-term functional and structural changes induced by magnetic stimulation (Rowan et al., 2018). Through cerebellar TMS, GABA-B receptor activation can induce alterations in the synthesis of GABA, the presynaptic GABA transporter, and cortical inhibitory interneurons, ultimately modulating the balance between excitation and inhibition within cerebellar circuits (Harrington and Hammond-Tooke, 2015). In this way, cerebellar TMS can facilitate neural plasticity and motor recovery, especially after stroke, where cerebellar inhibition is often impaired.

One of the key molecular pathways activated by cerebellar TMS is the BDNF pathway. BDNF regulates presynaptic GABA release (Song et al., 2022), which plays a pivotal role in synaptic plasticity, neuronal survival, and neurogenesis (Negrete-Hurtado et al., 2020). Cerebellar TMS has been shown to increase BDNF expression in the cerebellum and associated brain regions, thereby promoting the formation of new synapses, strengthening existing synapses, and supporting neuronal survival.

The receptor for BDNF, Tropomyosin Receptor Kinase B (TrkB), is activated during cerebellar TMS. The binding of BDNF to TrkB initiates a cascade of downstream signaling pathways that promote cellular survival, synaptic remodeling, and neuroplasticity. Furthermore, BDNF activation enhances synaptic efficiency and induces long-term alterations in neuronal connectivity, both of which are essential for motor learning and rehabilitation. Research by Mancic et al. (2016) has demonstrated that TMS interventions on the rat cerebellum can significantly affect the metabolic pathways of neuronal cells. In particular, TMS influences the expression of key metabolic enzymes and transporters, including glucose-6-phosphate dehydrogenase, vesicular glutamate transporter 1, plasma glutamate transporter 1, and glial fibrillary acidic protein. These metabolic alterations may support the energetic demands of neurons during the plasticity processes induced by TMS, further promoting functional recovery and synaptic plasticity.

## 3 Application of cerebellar TMS in stroke rehabilitation

A comprehensive literature search was conducted across several databases, including Web of Science, PubMed, Cochrane Library, and EMBASE, to identify relevant studies on cerebellar TMS. The search was executed by three reviewers (ZW, LikW, and FG) using the following terms: (cerebellum) AND (transcranial magnetic stimulation) AND (stroke) AND (movement). We also examined the references cited in the retrieved literature and the articles that cited these sources. The following criteria were used for cerebellar TMS studies: (1) clinical studies on stroke patients with impaired motor function, such as randomized controlled trials (RCTs) or case reports, etc.; (2) treatment groups received TMS; (3) published on peer-reviewed articles The exclusion criteria were as follows: (1) Duplicate reports; (2) Research protocols, conference abstracts, or incomplete studies; (3) Non-human research; and (4) Missing outcome information. This literature search was completed prior to March 1, 2024.

In our comprehensive review of current literature, we have identified 11 clinical trials and one case report predominantly focusing on the impact of cerebellar TMS on lower limb motor function and balance impairments post-stroke. Additionally, two singular study emphasized upper limb motor function (as detailed in Table 1). The majority of these studies encompass patients with subacute and chronic stroke, with the exception of three trials report that involved patients with acute stroke.

**Table 1:** Characteristics of the relevant studies.

Author/year	*N*	Experiment design	Movement disorder(s)	Target	Coil type (diameter/figure)	Coil placement/orientation	Intervention	Control	Behavioral training	Side effects	Main findings
Kim et al. (2014)	rTMS: sham (22: 10)	Pilot study	PCS with ataxia	Cerebellar hemisphere ipsilateral to the ataxic side (2 cm below and lateral to the middle line)	75 mm/8 coil by MagPro® (Medtronic, Minneapolis, MN, United States)	Manually placed/with the handle pointing superiorly	100% RMT 1 Hz rTMS (900 pulses per session, 5 sessions per day, 5 days)	Same parameters (coil perpendicular to the scalp)	None	None	1 Hz cerebellum rTMS, feasible and may have a beneficial effect in ataxic patients with posterior circulation stroke
Bonnì et al. (2014)	iTBS (6), no sham	Prospective pilot trial	Chronic stroke with PCS	The damaged lateral cerebellum	Monophasic Magstim 200 stimulator (Magstim Co., Whitland, Dyfeld, UK); 70 mm/8 coil	Neuronavigation system (Softaxic, E.M.S., Bologna, Italy)/The handle of the coil pointed backward and was perpendicular to the presumed direction of the central sulcus	80% AMT iTBS (600 pulses; 10 sessions, 2 weeks)	None	PT	None	Cerebellar iTBS could be a promising tool to promote recovery of cerebellar stroke patients
Koch et al. (2019)	iTBS: sham (18: 18)	RCT	Stroke with gait and balance impairment	Contralateral cerebellum to the affected cerebral hemisphere	70 mm/8 coil (Magstim Company)	Neuronavigation system (SofTaxic; EMS) coupled with a Polaris Vicra infrared camera/the handle pointing superiorly	80% AMT iTBS (1,200 pulses per session, 3 weeks)	Sham iTBS (coil perpendicular to the cerebellum)	PT	None	Cerebellar intermittent *θ*-burst stimulation promotes gait and balance recovery in patients with stroke by acting on cerebello-cortical plasticity
Xie et al. (2021)	iTBS: sham (17: 17)	Parallel group trial	Stroke with walking dysfunction	Contralateral cerebellum (1 cm inferior to and 3 cm lateral to the inion)	70 mm/8 coil (YIRUIDE medical, Wuhan, China)	Manually placed/the handle directed backward and laterally and at an angle of approximately 45° to the mid-sagittal line of the head	80% AMT iTBS (600 pulses per session, 10 sessions, 2 weeks)	Same parameters (coil pedicular to the scalp)	PT	None	iTBS over the contralesional cerebellum paired with physical therapy could improve walking performance in patients after stroke
Liao et al. (2021)	iTBS: sham (15: 15)	RCT	Stroke with balance and motor dysfunction	The cerebellar hemisphere contralateral to the affected cerebral hemisphere (3 cm lateral to the midline and 1 cm below the inion)	70 mm/8 coil (YIRUIDE medical, Wuhan, China)	manually placed/with the handle pointing superiorly	80% AMT iTBS (600 pulses per session, 10 sessions, 2 weeks)	Sham iTBS (coil angled at 90° to the scalp)	PT	Mild headache (*n* = 1) in the treatment group	Cerebellar iTBS associated with PT improves BBS score; no significant difference in FMA-LE scores; no difference in the BI scores
Li et al. (2021)	rTMS + cTBS: rTMS: cTBS (30: 30: 30)	RCT	Stroke with spasticity and limb dyskinesia	M1 on the unaffected side of the brain; right cerebellar hemisphere (3 cm lateral to the midline and 1 cm below the inion)	Circular coil	Manually placed/none	80% AMT cTBS (1,200 pulses); 80% RMT 1 Hz rTMS (1,000 pulses); 1 session per day, 6 sessions per week, 4 weeks	Only cTBS; only LF-rTMS	PT + acupuncture therapy	Not to mention	The MAS score was markedly decreased; FMA and MBI scores were markedly increased in the three groups; LF-rTMS + cTBS group showed lower MAS score, higher FMA, and MBI scores
Chen et al. (2021)	iTBS: sham (16: 16)	RCT	Stroke with upper limb spasticity	Ipsilesional lateral cerebellum (1 cm inferior and 3 cm lateral to the inion)	70 mm/8 coil (YIRUIDE medical, Wuhan, China)	Manually placed/with the handle pointing superiorly	80% AMT iTBS (600 pulses per sessions, 10 sessions, 2 weeks)	Same parameters (coil was rotated 90°)	PT	None	Both groups showed significant improvements in the MAS, MTS, SWV, and BI. Compared with the sham stimulation group, MAS, MTS, SWV, and MEP amplitude are improved more in the cerebellar iTBS group
Rosso et al. (2022)	CER_M1 PAS: sham (14: 14)	RCT	Stroke with upper limb dysfunction	The coil was moved right- or left- wards off the midpoint by 3 cm along a line between the inion and the mastoid process	110 mm/double-cone coil; 70 mm/8 coil	By the anatomical 3D reconstruction of each participant’s brain (Brainsight2, Rogue Research, Inc., Montreal, Canada)/none	PAS (120 pairs of stimuli at 0.2 Hz) (Magstim, Dyfed, UK); conditioning stimulus: 90% RMT; test stimulus:140% RMT; 5 sessions, 1 week	same place (sham coil produces a sound mimicking)	PT	transient cephalalgia (*n* = 1) in each group; reflex syncope right (*n* = 1) in the sham group	Cerebello-motor PAS was effective compared to sham in improving hand dexterity but not grip strength
Im et al. (2022)	rTMS:control (16:16)	Placebo-controlled study	Cerebral infarction with balance impairment	2 cm below and 2 cm lateral to the inion by targeting the cerebellar hemisphere contralateral to the site of cerebral infarction	8 coil Magpro R30 (Magventure, Farum, Denmark)	Manually placed/none	90% RMT 1 Hz (900 pulses)	Sham coil (the sound and scalp sensation were similar)	PT	vertigo (*n* = 1) in the treatment group	Low-frequency cerebellar rTMS is helpful for improving balance in patient with cerebral infarction, and maybe a beneficial treatment for these patients
Xia et al. (2022)	CB-single iTBS: CB-M1 iTBS:CB-SMA iTBS (9:10:11)	Pilot study	Stroke with motor and balance defects	M1: motor hot spot; SMA: 3 cm in front of Cz and 0.5 cm close to the hemisphere; cerebellum stimulation point: 1 cm below and 3 cm lateral to the inion	90 mm/8 coil (YIRUIDE medical, Wuhan, China)	Manually placed/none	3 pulses at 50 Hz repeated at 5 Hz (600 pulses in 192 s)	None	None	None	The CB-SMA group exhibited a significant inhibitory pattern in the resting-state functional connectivity, which was not observed in the other two groups. In conclusion, we believe that paired targeting of the CB-SMA can reshape the brain network and improve the balance function of patients with stroke
Einstein et al. (2023)	None	Case report	Hemorrhagic stroke with chronic nonlateralized ataxia	Left: 2 cm laterally from the midline and approximately 1 cm below the inion. Right: 2 cm laterally from the midline and 2 cm below the inion	B-70 coil (Magventure, Denmark)	Using a Brainsight system (Rogue Research, Montreal, Canada) that was then used to guide stimulation/none	100%MT 1 Hz rTMS (900 pulses, 5 sessions per day, 2 days)	None	None	None	The case of a patient with chronic cerebellar ataxia following a hemorrhagic stroke who underwent inhibitory rTMS to bilateral cerebellar targets with demonstrated improvement in symptoms
Liao et al. (2024)	M1 iTBS: cerebellum iTBS: sham (12: 12: 12)	RCT	Stroke with motor and balance defects	Contralateral cerebellar hemisphere (3 cm lateral to the midline and 1 cm below the inion of the occipital bone); ipsilesional M1	70 mm/8 coil (YIRUIDE medical, Wuhan, China)	Manually placed/with the handle pointing superiorly	80% RMT 50 Hz iTBS, (1,200 pulses per session, 15 sessions, 3 weeks)	Sham stimulation (coil was rotated 90°)	PT	None	Stimulation of the cerebellum and M1 both improves BBS score and change FAC scores. Cerebellar stimulation may be better than stimulation of the M1 since the changes in the BBS scores in the cerebellar-iTBS group seemed to be larger than M1-iTBS group although there was no statistical significance

RCT, randomized controlled trial; PAS, paired associative stimulation; PCS, posterior circulation stroke; CB-single, unilateral cerebellar; CB-M1, cerebellar–primary motor cortex; CB-SMA, cerebellar–supplementary motor area; AMT, the active motor threshold; MT, motor threshold; RMT, resting motor threshold; PT, physical therapy; FMA, FMA-LE scores; MAS, the modified Ashworth scale; MBI, modified Barthel index; MTS, the modified Tardieu scale; SWV, the shear wave velocity; MEP, motor-evoked potential; BBS, Berg balance scale; BI, the Barthel index.

The array of methodologies in cerebellar TMS, ranging from low-frequency rTMS to iTBS and cTBS, supplemented by advanced techniques such as Cerebello-Motor Paired Associative Stimulation (PAS), presents a diverse and adaptable toolkit for stroke rehabilitation.

### 3.1 Low-frequency rTMS

Low-frequency rTMS is characterized by its inhibitory effects on cerebellar activity and has demonstrated efficacy in alleviating ataxia and balance disorders symptoms. For instance, Kim et al. (2014) applied this technique in patients with posterior circulation stroke and observed significant improvements in gait dynamics and balance. The enhancements noted in the 10-Meter Walk Test (10MWT) and Berg Balance Scale (BBS) scores translated into substantial gains in daily activities and mobility.

The application of low-frequency rTMS to the cerebellum primarily works by modulating cerebellar-brain inhibition (CBI), a process that regulates how the cerebellum communicates with the M1 (Im et al., 2022; Einstein et al., 2023). By inhibiting excessive cerebellar excitability, low-frequency rTMS helps recalibrate overactive cerebellar output, commonly seen in conditions like ataxia. This modulation likely reduces the disruptive effects of impaired cerebellar signals on motor coordination, allowing for more controlled movement patterns (Lien et al., 2022). Additionally, rTMS may promote plastic changes in cerebello-cortical pathways, enhancing the reorganization of motor networks that are crucial for motor recovery post-stroke.

### 3.2 Intermittent TBS

The utilization of iTBS targeting the cerebellum is predominantly applied in addressing limb dysfunction following a stroke. Xia’s review (Xia et al., 2023) posits that the effectiveness of cerebellar stimulation is not attributable to a single session, but rather to the cumulative impact of TMS. This was later substantiated by further research conducted by Xia et al. (2022). In the study conducted by Bonnì et al. (2014), iTBS was directed toward the impaired lateral cerebellum in patients with chronic stroke, resulting in heightened excitability of the cerebellar cortex. This approach led to notable enhancements in both the subacute and chronic stages of stroke rehabilitation.

Building on this, Koch et al. (2019) provided evidence for the efficacy of iTBS in improving balance and gait functions. This was demonstrated through significant improvements in various assessments including the BBS, Scale Trunk Impairment Scale, Fugl-Meyer Assessment-Lower Extremity, and the Melbourne Assessment of Unilateral Upper Limb Function. These improvements collectively indicate advancements in balance, trunk control, lower limb motor functionality, and ataxia. Moreover, Xie et al. (2021) observed that integrating iTBS with physical therapy notably enhanced walking performance in stroke patients.

The primary mechanism through which iTBS enhances motor recovery lies in its ability to induce LTP-like effects in the cerebellar cortex. By increasing the excitability of cerebellar networks, iTBS promotes synaptic plasticity, facilitating the reorganization of damaged motor circuits and enhancing communication between the cerebellum and M1 (Chen et al., 2019; Hensel et al., 2019). This improved connectivity strengthens motor coordination and learning, which is especially critical in post-stroke recovery.

### 3.3 Continuous TBS

CTBS, akin to low-frequency rTMS, has shown particular efficacy in combined therapy approaches. Li et al. (2021) demonstrated that when combined with low frequency rTMS (LF-rTMS), cTBS contributed to significant reductions in muscle spasticity and improvements in limb dyskinesia, offering a synergistic benefit surpassing individual treatments.

The effectiveness of cTBS lies in its ability to induce LTD-like effects (Romero et al., 2022) in the cerebellum, which inhibits excessive neural excitability. This downregulation of overactive motor circuits contributes to the reduction of spasticity and dyskinesia by restoring a more balanced and controlled output from the motor cortex. When combined with LF-rTMS, which exerts an inhibitory effect on hyperexcitable neural circuits in the motor cortex, this dual therapy effectively targets both cortical and cerebellar pathways. This approach enhances the neuroplastic changes essential for improving motor control, particularly in conditions characterized by increased muscle tone and involuntary movements.

### 3.4 Cerebello-motor paired associative stimulation

By combining TMS with peripheral nerve stimulation, PAS facilitates spike-timing-dependent plasticity, targeting either cortical–cortical (C/C PAS), cortical-peripheral (C/P PAS), or even cerebellar-motor (Cerebello-M1 PAS) connections to strengthen neural pathways involved in motor control. Stimulating both the M1 and the cerebellum simultaneously can further enhance the reorganization of motor networks, offering an additional pathway to improve motor outcomes.

The targeting of paired sites within the cerebellum and cerebral cortex may yield more advantageous outcomes compared to stimulating individual sites. Xia’s investigation (Xia et al., 2022) into the effects of a single TMS session on balance, with eyes open and closed, in stroke patients utilized three distinct stimulation targets: unilateral cerebellum, cerebellar-M1, and cerebellar-SMA. The findings indicated that combined targeting of the cerebellum and SMA facilitated the restructuring of brain networks, leading to improved balance functions in these patients. Rosso et al. (2022) investigated the efficacy of Cerebello-Motor PAS, revealing its effectiveness in improving hand dexterity compared to sham interventions. This technique, which synergizes cerebellar and cortical stimulation, employs associative plasticity principles with the objective of bolstering functional connectivity between the cerebellum and motor cortex, potentially facilitating cortical reorganization conducive to motor recovery.

### 3.5 Integration with physiotherapy

The integration of cerebellar TMS techniques with traditional physiotherapy has proven more effective than isolated interventions. Liao et al. (2021) combined iTBS with physical therapy, leading to improvements in BBS scores, indicative of enhanced balance and motor recovery. Similarly, Chen et al. (2021) found that iTBS could augment the effects of physical therapy in addressing upper limb spasticity post-stroke. This collaborative approach, melding neural modulation with physical therapy, hints at a more holistic model of rehabilitation. This model is further supported by Liao’s comparative study (Liao et al., 2024), which suggested that cerebellar stimulation might surpass M1 iTBS in facilitating balance and motor recovery.

The neuroplasticity mechanisms underlying this combined application of cerebellar TMS and physical therapy are rooted in both specific and generalized processes. A key specific mechanism is spike-timing-dependent plasticity (STDP) (Bi and Poo, 2001; Dan and Poo, 2006), which occurs when the timing of TMS pulses is aligned with motor or sensory input from physical therapy, leading to the targeted strengthening or weakening of synaptic connections (Rosenkranz et al., 2014). This precise timing enhances corticomotor excitability and strengthens neural circuits critical for motor learning and recovery.

## 4 Predictors of response to cerebellar TMS in stroke rehabilitation

However, not all patients respond equally to cerebellar TMS, and identifying predictors of response is essential for optimizing treatment outcomes. These predictors can help guide clinicians in tailoring cerebellar TMS protocols, such as stimulation intensity, frequency, and target sites, according to individual patient characteristics.

Predictors of response to cerebellar TMS encompass a range of biological, neurophysiological, neuroimaging, genetic, and clinical markers that can influence how effectively cerebellar stimulation facilitates motor and cognitive recovery. Unlike TMS targeting the M1, cerebellar TMS engages distinct neural circuits and mechanisms, such as CBI and cerebello-cortical connectivity, which may necessitate unique predictors to accurately forecast treatment success. For instance, neurophysiological markers like cerebellar-specific MEPs and neuroimaging indicators of cerebello-cortical connectivity provide insights into the integrity and plasticity of cerebellar pathways, directly influencing the responsiveness to cerebellar TMS (Koch et al., 2019; Tan et al., 2021).

### 4.1 Motor evoked potentials in cerebellar stimulation

The cerebellum’s influence on motor cortex excitability has garnered significant interest in neurophysiology and rehabilitation, particularly in understanding how cerebellar stimulation can enhance Motor Evoked Potentials (MEPs), which are critical indicators of corticospinal tract functionality and neuroplasticity. Studies utilizing cerebellar TBS have revealed its significant effects on M1 excitability and intracortical dynamics, which can serve as potential predictors of motor recovery.

Koch et al. (2008) demonstrated that cTBS applied to the lateral cerebellum leads to a decrease in short-interval cortical inhibition (SICI) and an increase in long-interval cortical inhibition (LICI), while iTBS demonstrates the opposite effect, increasing M1 excitability. These changes in MEPs indicate how different patterns of cerebellar stimulation can modulate motor cortex activity, offering insights into which stimulation protocols may yield better recovery outcomes for specific patients. Spampinato D. et al. (2020) further investigated how cerebellar-M1 networks are activated by different TMS pulse orientations, suggesting that distinct neural networks are engaged based on stimulation parameters, contributing uniquely to motor recovery.

Additionally, Pauly et al. (2021) examined the effects of rTMS and PAS on cerebellar plasticity in healthy individuals, showing that rTMS at 1 Hz facilitates cerebellar-M1 interactions, as evidenced by increased MEP amplitudes and higher motor thresholds. Conversely, PAS produced inhibitory effects, characterized by decreased MEP amplitudes, suggesting that the type of stimulation directly impacts how motor circuits are modulated. Bonnì et al. (2014) observed that iTBS applied to the cerebellum induced changes in CBI and intracortical facilitation (ICF), which were paralleled by clinical improvements in motor function.

These findings collectively highlight the dynamic role of cerebellar stimulation in modulating MEPs and their potential as predictors of response to TMS in stroke rehabilitation. By monitoring changes in MEPs following cerebellar TMS, clinicians can better assess the likelihood of motor recovery and adjust treatment protocols accordingly.

### 4.2 Neuroimaging predictors: brain activity and connectivity in cerebellar TMS response

In stroke rehabilitation, the activity of specific brain regions and the connectivity between them have emerged as promising predictors of response to cerebellar TMS. Neuroimaging tools such as Functional Magnetic Resonance Imaging (fMRI) and Functional Near-Infrared Spectroscopy (fNIRS) are critical in assessing these neural patterns, helping to identify patients who are more likely to benefit from cerebellar stimulation.

The activation of motor-related brain regions, particularly the M1, can serve as a key indicator of how well a patient may respond to cerebellar TMS. Studies using fMRI have shown that increased activity in the ipsilesional M1 following cerebellar stimulation correlates with improved motor function. For example, Rosso et al. (2022) demonstrated that patients with greater post-stimulation activation in M1 exhibited better hand dexterity recovery. This suggests that the level of motor cortex activation could act as a biomarker for predicting motor improvements in response to cerebellar TMS.

Additionally, changes in activity within other motor-related areas, such as the premotor cortex and supplementary motor area (SMA), could also provide predictive value. These regions are involved in motor planning and coordination, and their engagement during rehabilitation may indicate how well a patient can adapt to and benefit from stimulation therapies.

Beyond the activity of individual brain regions, the functional connectivity between brain regions can also serve as a powerful predictor of TMS response. fMRI and fNIRS can measure the strength of connections between the cerebellum and cortical motor areas, such as M1 and the SMA, providing insights into the brain’s network-level adaptations. For instance, Xia et al. (2022) used fNIRS to explore how different iTBS protocols affected connectivity between the cerebellum and motor networks. They found that specific protocols, such as cerebellum-SMA stimulation, produced significant connectivity changes that correlated with improvements in balance and motor control. This suggests that enhanced connectivity between motor regions may serve as a predictor of positive outcomes in response to cerebellar TMS.

By using the activity of specific brain regions and the connectivity between them as predictors, clinicians can tailor cerebellar TMS protocols to target the most responsive areas of the brain. This approach can help optimize rehabilitation strategies for stroke patients by focusing on regions and networks that are most likely to contribute to motor and balance recovery. Neuroimaging techniques such as fMRI and fNIRS offer valuable tools for monitoring these neural markers, enabling personalized and effective treatment plans.

### 4.3 Cognitive predictors in cerebellar TMS response

When investigating the effects of cerebellar TMS in noninvasive studies, it is essential to consider potential cognitive confounders, as cognitive processes are intricately linked with motor learning and recovery (Hardwick et al., 2021). While much of the existing research on cerebellar TMS and cognition has been conducted in healthy individuals, we believe that these findings can still be relevant to stroke rehabilitation. Cognitive processes, such as working memory, attention, and error monitoring, are critical to motor learning and the reacquisition of motor skills post-stroke (Liu et al., 2022). These cognitive functions, although less frequently studied in stroke patients, may serve as important predictors of a patient’s response to cerebellar TMS.

The cerebellum’s role extends beyond motor coordination; specific regions, such as Crus I and II, are implicated in cognitive functions like the perception of emotional states and interaction with prefrontal cognitive areas (Ramnani, 2006; Strick et al., 2009). Neuroimaging studies have revealed that cerebellar activation patterns correlate with enhanced cognitive processing and improved motor learning outcomes (Riedel et al., 2015). Casula et al. (2016) demonstrated that cerebellar TMS can modulate neural activity in motor-cognitive networks, with cTBS reducing alpha activity and intermittent iTBS enhancing beta activity in the M1. This bidirectional modulation highlights the potential for cerebellar TMS to influence cognitive processes. Although these studies have largely focused on healthy populations, the cognitive-motor interactions identified may also apply to stroke recovery, where cognitive impairments can impact the success of motor rehabilitation.

Cognitive functions, closely intertwined with motor abilities, may serve as predictors of response to cerebellar TMS. Evidence from healthy individuals suggests that enhancing cognitive processes through cerebellar TMS can improve task performance and decrease error rates in motor learning tasks (Matsugi et al., 2022). Although data specific to stroke patients are limited, it is plausible that those with stronger baseline cognitive function may respond better to cerebellar TMS interventions aimed at improving motor skills. Moreover, cerebellar TMS has been shown to expedite learning in tasks such as force field and locomotor adaptation (Celnik, 2015). These enhancements are likely mediated by cognitive processes essential to motor adaptation, further underscoring the role of cognition in rehabilitation. Given the cerebellum’s involvement in both motor and cognitive networks, it is feasible to speculate that cognitive abilities may predict how well a patient responds to TMS in stroke rehabilitation.

Future studies should investigate the role of cognition in cerebellar TMS response more directly in stroke patients, given the potential for cognitive functions to serve as predictors of motor rehabilitation outcomes.

## 5 Challenges and future directions

Despite the potential of cerebellar TMS in stroke rehabilitation, its application is met with several challenges and limitations, which necessitate future exploration and refinement.

### 5.1 TMS coil type

Whether differences in the type of TMS coil used significantly affects treatment outcomes is uncertain. Most cerebellar TMS studies (Bonnì et al., 2014; Kim et al., 2014; Koch et al., 2019; Chen et al., 2021; Li et al., 2021; Liao et al., 2021; Xie et al., 2021; Im et al., 2022; Einstein et al., 2023; Liao et al., 2024) have utilized flatter coils, such as figure-eight or circular coils, offering a spatial resolution of about 1 cm and a penetration depth of approximately 2 cm (Li et al., 2019). While the figure-eight coil provides more focused stimulation than the circular coil, its depth and intensity are inferior to that of angled coils (Deng et al., 2013; Drakaki et al., 2022). The cerebellum, situated in the posterior cranial fossa and covered by the tentorium cerebelli, is deeper from the skull, necessitating coils that can accommodate this depth. Previous research has indicated that while flat coils improve stimulation tolerance, they are limited in depth range, and smaller coils are less effective for cerebellar stimulation (Spampinato D. et al., 2020). Therefore, angled coils with superior depth properties are essential for reliable CBI excitation (Fernandez et al., 2018). Innovations in coil design, such as the use of a biconical coil, have shown promise. Rosso et al. (2022) demonstrated significant improvements in hand dexterity in stroke patients using cerebellar motor paired associative stimulation with a 110 mm biconical coil combined with physical therapy. This coil type can stimulate a depth of 3–4 cm, activating specific GABA-dependent interneurons in the cerebellum and enhancing LTP effects (Lu and Ueno, 2017). Currently, the MEP amplitude produced by biconical coils is believed to be higher than that of figure-eight coils under the same stimulation intensity, making it more effective for cerebellar stimulation at tolerable intensities (Liao et al., 2021; Xue et al., 2021). However, there is a risk of stimulating non-target functional areas, which may reduce patient tolerance (Fernandez et al., 2018; Xie et al., 2021).

In summary, the challenges in cerebellar TMS primarily revolve around the optimization of coil types and stimulation parameters. Future research should focus on developing and testing coils that offer the right balance between depth of penetration, focus of stimulation, patient tolerance, and therapeutic efficacy. Such advancements could significantly enhance the effectiveness of cerebellar TMS in stroke rehabilitation and potentially in other neurological disorders.

### 5.2 Safety and tolerability

The 2020 TMS Use Guidelines emphasize seizures as the primary risk in TMS across different stimulation methods. Regarding cerebellar TMS specifically, side effects have been infrequently reported in the literature (Table 1). In 11 studies using flat coils, one noted a mild headache in the treatment group and reflex syncope in an individual in the sham group (Liao et al., 2021), while another recorded a case of vertigo in the treatment group (Im et al., 2022). A singular study employing a conical coil reported transient cephalalgia in one participant each in the intervention and non-intervention groups (Rosso et al., 2022). Notably, five patients in the active group and two in the sham group reported discomfort post-intervention. In a recent study by Dai et al. (2023), a biconical coil was used to assess the efficacy of 10-Hz cerebellar rTMS in patients with poststroke dysphagia who had suffered infratentorial strokes. The study demonstrated that all 42 participants successfully tolerated the treatment. However, this transient twitching was reported by patients undergoing both bilateral (14 patients) and unilateral (8 patients) cerebellar rTMS, as well as one patient receiving sham rTMS, which resolved quickly after each session. Notably, one individual noted a gradual increase in the intensity of the twitching sensation over the course of the treatment. Additionally, a thorough meta-analysis on the safety of cTBS included 45 studies (Hurtado-Puerto et al., 2020), none of which reported severe adverse events. The withdrawal rate due to adverse events was only 0.72%. However, the maximum safe dosage for cerebellar TMS remains undefined. Consequently, more research to determine the maximum safe dose of cerebellar TMS is crucial for safeguarding patient well-being and enhancing therapeutic effectiveness.

### 5.3 Optimal timing for stimulation

To date, research has yet to establish the ideal time frame for commencing treatment of cerebellar TMS following a stroke. Eline’s meta-analysis (van Lieshout et al., 2019) suggests that rTMS may offer greater benefits when initiated within the first month after a stroke. Although many clinical practice guidelines recommend the early start of rehabilitation post-stroke, these studies focus on patients in the subacute and chronic phases of stroke recovery (Bonnì et al., 2014; Koch et al., 2019; Chen et al., 2021; Li et al., 2021; Liao et al., 2021; Rosso et al., 2022; Xia et al., 2022). However, there are exceptions, as three RCTs (Kim et al., 2014; Xie et al., 2021; Liao et al., 2024) have reported on patients in the acute stage of stroke, indicating the potential applicability of cerebellar TMS across various stages post-stroke.

### 5.4 Stimulation modes and targets

In the field of cerebellar research, the majority of cerebellar TMS parameters are adapted from those used for cerebral cortex stimulation. Presently, there is an absence of standardized protocols specifically tailored for effective cerebellar stimulation. Common targets for cerebellar stimulation for patients, a typical approach involves positioning the stimulation site 3 cm lateral to the occipital tuberosity and then moving it 1 cm downward. This is particularly challenging given the higher complexity and deeper distribution of the cerebellar cortex compared to other brain regions (Hardwick et al., 2014). Because it is difficult to stimulate motor regions of the cerebellum without also stimulating the cognitive cerebellum. These anatomical variances among individuals can significantly influence the response to cerebellar stimulation, potentially leading to less effective outcomes compared to stimulation of cerebral targets (Chung et al., 2016). Excessively low stimulation intensities may be less efficacious, while overly high intensities could diminish patient compliance and elevate the risk of adverse effects (Fernandez et al., 2018). Moreover, the individual preservation of neural network structure and the integrity of efferent pathways are crucial factors in customizing stimulation patterns (Hardwick et al., 2021). This highlights the ongoing need to explore and identify more appropriate cerebellar stimulation sites for future research.

The mechanisms through which cerebellar TMS enhances function in stroke patients vary according to the injury’s location. Nevertheless, the existing literature on this topic remains limited, highlighting the need for a more detailed examination of the specific disease locations (Dai et al., 2023). In supratentorial strokes, cerebellar TMS primarily enhances motor recovery by leveraging the cerebellum’s role in modulating cortical activity. The cerebellum communicates with both the motor and premotor areas of the cortex (Wessel and Hummel, 2018), and TMS applied to the cerebellum can increase the excitability of motor pathways, improving motor control and coordination. This can help in restoring motor function by compensating for cortical damage. In contrast, for cerebellar or brainstem injuries, TMS likely works by promoting neuroplasticity within the cerebellum and its connections to the rest of the brain. The cerebellum’s extensive connections with the brainstem, spinal cord, and thalamus can facilitate compensatory reorganization in these areas (Li et al., 2018; Tan et al., 2021). There are numerous methods to stimulate the cerebellum; however, accurately locating it and understanding the heterogeneity caused by the disease’s location still require further investigation.

## 6 Limitations of this review

Reviews based on high-quality RCTs are essential for clinical decision-making in evidence-based medicine. However, the limited number of included studies and considerable clinical heterogeneity—such as varying TMS treatment regimens and combination approaches—pose challenges for this review. The study primarily employed qualitative analysis through narrative reviews instead of quantitative methods like meta-analysis. Additionally, it is important to acknowledge that quality assessment remains a subjective process. The article underwent review by three independent reviewers, who may have made differing judgments on each factor, potentially leading to variations in the results. Lastly, due to resource constraints, only studies published in English were included, which may introduce a language bias and exclude relevant literature published in other languages.

## 7 Conclusion

In conclusion, cerebellar TMS emerges as a promising therapeutic modality in the rehabilitation of post-stroke limb dysfunction. The research reviewed underscores its potential to target deeper motor areas, manage lower limb and balance dysfunctions, and improve motor and cognitive aspects of stroke recovery. While challenges related to coil design, safety, and stimulation parameters remain, ongoing research and technological advancements hold promise for more refined, effective treatments. The future of cerebellar TMS in stroke rehabilitation is poised for significant growth, particularly as our understanding of cerebellar functions in motor control and learning deepens. To fully harness the potential of cerebellar TMS, further research should focus on optimizing stimulation protocols, understanding individual variability in responses, and integrating TMS with other rehabilitation strategies. This holistic approach could revolutionize stroke rehabilitation, enhancing the quality of life for countless individuals affected by this debilitating condition.
